# Doing our best and doing no harm: A focused ethnography of staff moral experiences of providing palliative care at a Médecins Sans Frontières pediatric hospital in Cox’s Bazar, Bangladesh

**DOI:** 10.1371/journal.pone.0288938

**Published:** 2023-07-20

**Authors:** Rachel Yantzi, Md Hadiuzzaman, Pradip Kumar Sen Gupta, Amin Lamrous, Kathryn Richardson, John Pringle, Lisa Schwartz, Puspita Hossain, David Kizito, Sakib Burza

**Affiliations:** 1 Médecins Sans Frontières, Cox’s Bazar, Bangladesh; 2 Department of Health Research Methods, Evidence & Impact, McMaster University, Hamilton, Canada; 3 Department of Epidemiology, Bangladesh University of Health Sciences, Dhaka, Bangladesh; 4 Médecins Sans Frontières, Barcelona, Spain; 5 Faculty of Infectious and Tropical Diseases, London School of Hygiene and Tropical Medicine, London, United Kingdom; Jhpiego, UNITED STATES

## Abstract

**Introduction:**

The Médecins Sans Frontières (MSF) Goyalmara Hospital in Cox’s Bazar, Bangladesh is a referral centre offering the highest level of care available in the Rohingya camps for pediatrics and neonatology. Efforts are underway to integrate pediatric palliative care due to high mortality and medical complexity of patients, yet little is known about the experiences of staff delivering palliative and end-of-life care. The purpose of this study was to understand the moral experiences of MSF staff to inform program planning and implementation.

**Methods:**

This focused ethnography was conducted between March-August 2021 at Goyalmara Hospital. Data collection involved participant-observation, individual interviews (22), focus group discussions (5), and analysis of documents including MSF clinical guidelines, admission and referral criteria, reports, and training materials. Data analysis followed a modified version of the Qualitative Analysis Guide of Leuven and data were coded using NVivo software.

**Results:**

The prevailing understanding of pediatric palliative care among national and international staff was care that prioritized comfort for infants and children who were not expected to survive. Staff’s views were informed by their sense of obligation to do no harm, to do their best on behalf of their patients, and religious beliefs about God’s role in determining the child’s outcome. The authority of doctors, international staff, as well as protocols and guidelines shaped palliative care decision-making. Staff saw clinical guidelines as valuable resources that supported a consistent approach to care over time, while others were concerned that palliative care guidelines were rigidly applied.

**Conclusion:**

When integrating palliative care into humanitarian programs, it is important to emphasize the active role of palliative care in reducing suffering. Advocacy for access to the highest level of care possible should continue alongside palliative care integration. While palliative care guidelines are valuable, it is essential to encourage open discussion of staff concerns and adapt care plans based on the family’s needs and preferences.

## Introduction

Palliative care aims to prevent and relieve physical, psychological, social, and spiritual suffering among adults, children, and families facing life-threatening illness [1] through the “early identification and impeccable assessment and treatment” of suffering [1]. Despite increased awareness of palliative care globally, access to pediatric palliative care lags behind access to care for adults, particularly in low and middle income countries (LMICs) where 98% of children with palliative care needs reside [2]. Humanitarian organizations provide assistance to populations in distress in contexts where conflict, forced displacement, disasters, or disease outbreaks have led to massive loss of life and disruption of mechanisms by which communities care for one another [1, 3]. As a result, the need for palliative care during humanitarian crises is particularly acute [1, 4]. While medical humanitarian organizations have historically focused their efforts on saving lives, palliative and end-of-life care for both adults and children are increasingly recognized as important components of the humanitarian mandate to alleviate suffering and restore dignity [1, 4, 5]. Growing attention to palliative care in humanitarian response is evidenced by the 2018 publication of the World Health Organization (WHO) guide, *Integrating palliative care and symptom relief into the response to humanitarian emergencies and crises* [1], and the addition of palliative care to the SPHERE standards for humanitarian response [5]. Yet humanitarian health professionals and policy makers report a number of barriers have that have slowed the integration of palliative care in humanitarian response including an ethos that prioritizes life-saving care, concerns about the allocation of scarce resources, concerns about public perceptions and the impact on fundraising, absence of guidelines and technical expertise, barriers to accessing opioid pain medications, and the cultural specificity of death and dying [6].

Moral distress has been acknowledged as a significant problem among humanitarian healthcare providers [7, 8], and the literature indicates that failing to address the suffering of adults and children with palliative care needs may lead to moral distress for humanitarian healthcare providers [9] Moral distress is most commonly understood as a stress reaction to situations where a person feels unable to fulfill their moral values [8], and it can have a variety of consequences including anger, guilt, diminished confidence, depression, anxiety, and burn-out [8, 10]. Moral distress that is chronic and unresolved may diminish humanitarian healthcare workers’ ability to act in accordance with their values, connect on an emotional level with their patients, and provide quality care [8, 11]. Moral experience is a related but different concept that may incorporate moral distress but goes further to include a person or group of peoples’ lived experiences of situations that fall anywhere on the spectrums of right-wrong, good-bad or just-unjust [12]. Given the ethical complexity of palliative care in humanitarian response and negative impacts of moral distress, the moral experiences of healthcare providers are likely to impact their reactions to palliative care program implementation efforts, the feasibility of those programs, and healthcare providers’ ability to offer effective, empathetic, and person-centred palliative and end-of-life care.

In recent years, the medical humanitarian organization Médecins Sans Frontières (MSF) has taken steps to incorporate palliative and end-of-life care in several projects in Guinea-Bissau, India, South Sudan, and Bangladesh [13, 14]. MSF operational research activities have begun to explore healthcare provider and patients’ perceptions of palliative care including a mixed-methods study of healthcare provider knowledge, attitudes, and practices regarding palliative care in an advanced HIV project in Patna, India. This study found that knowledge of palliative care among staff was fairly limited, and communication about prognosis with terminally ill patients was a challenge [15]. Another qualitative study of international staff returning after an assignment with MSF found that participants experienced frustration and distress related to their inability to adequately treat pain among patients living with non-communicable disease [16]. Since the formal inclusion of palliative care in humanitarian response is relatively new, very little is known about the experience of staff involved in these programs.

### The Rohingya refugee crisis and study context

The Rohingya people have faced decades of oppression, genocide, and statelessness in Myanmar leading to repeated influxes of Rohingya refugees fleeing across the border into Bangladesh [17–19]. The current Rohingya refugee crisis in Cox’s Bazar District was precipitated by violence targeting Rohingya communities in Rakhine state, Myanmar in August 2017, with approximately 919,000 Rohingya refugees displaced into overcrowded camps in Cox’s Bazar District, Bangladesh [20]. The humanitarian response in Cox’s Bazar, including efforts to integrate palliative care, has been complicated by the lack of freedom of movement for Rohingya refugees, restricted humanitarian access to the camps, high turnover of locally recruited staff as experienced nurses, doctors and midwives leave the NGO sector to take positions in the Bangladeshi government system, mistrust of healthcare workers, and growing hostility between the host community and Rohingya population [21–23]. The trauma and loss that Rohingya refugees have experienced, policies that exclude them from employment and education, and the lack of resolution of the displacement crisis have contributed to widespread psychological distress and growing despair within the Rohingya community in Bangladesh [22, 24].

A 2017 cross-sectional study conducted in Cox’s Bazar district revealed significant unmet palliative care needs among Rohingya refugees living with chronic or life-threatening illness [25]. For example, 70.5% of participants living with serious health problems (n = 156) reported experiencing moderate or severe pain in the past 3 days, and 59.1% of participants who sought care in a health facility reported that treatment was ineffective at addressing their presenting complaint [25]. While this study was conducted shortly after the main influx of Rohingya refugees and palliative care needs may have changed, the 2020 Joint Response Plan for the Rohingya Humanitarian Crisis acknowledged that non-communicable disease care, including services for people with disabilities and palliative care, are “not adequate to the needs” [26]. In 2021, the only other agencies offering palliative care services in the camps were Health Management in Broader Dimension (HMBD) Foundation, a small Bangladeshi non-governmental organization (NGO) [27], and a collaborative program between the International Organization on Migration (IOM) and the Fasiuddin Khan Research Foundation (FKRF) [28, 29]. In January 2021, an MSF team visited both programs to assess palliative care capacity and referral options in the camps. At that time, the HMBD program offered outpatient and home-based care services in Camp-13 and was staffed by one physician and three palliative care assistants (trained lay health workers) (RY fieldnotes). The IOM program offered outpatient and home-based care palliative care services at five Primary Health Care Centres (PHCCs) inside the camps [29]. Team composition varied by site but generally included 1 nurse, 1 palliative care assistant, 1–2 doctors, and a physiotherapist. Unfortunately, COVID-19 negatively impacted the ability of both programs to offer home-based care, and at the time of our site visit the IOM/FKRF program was primarily offering outpatient services (RY field notes). Coverage of these programs was limited to 5 of the 34 camps in Cox’s Bazar district and care was not accessible for the Bangladeshi host community [26].

In response to healthcare needs identified in Cox’s Bazar, MSF operates Goyalmara Mother-Child Hospital, a referral centre offering the highest level of pediatric and neonatal care available in the Rohingya camps. Goyalmara receives patients referred from throughout Ukhiya and Teknaf upazillas (sub-districts), which have a population of approximately 481,000 Rohingya and 286,000 Bangladeshi children under the age of 18 [20]. Between January and July 2021, an average of 64 children and 109 neonates were admitted per month to the Pediatric Intensive Care Unit (PICU) and neonatology department respectively (unpublished administrative data). Care in the intensive care units is limited to non-invasive ventilatory support and advanced organ support is absent. As a result, neonatal mortality in particular is high due to the medical complexity of the patient population and limited referral options. For example, Neonatal Intensive Care Unit (NICU) mortality between January to May 2021 varied between 13–21% and PICU mortality during that same period ranged between 6–12% [30]. Significant efforts were made to improve the quality of care and reduce inpatient mortality including the implementation of non-invasive ventilatory support (CPAP) in the NICU and PICU, resuscitation and nursing care training, and an audit to understand factors contributing to high rates of perinatal asphyxia among neonates referred to Goyalmara. Integration of palliative and end-of-life care was understood to be another important aspect of improving the quality of care. Given the high neonatal mortality and recognition that many of these deaths were unavoidable in the context, project leadership made the decision to begin palliative care activities with a focus on end-of-life care with plans to later expand the focus to children with chronic palliative care needs.

Palliative care was first introduced at Goyalmara Hospital in 2019 during a brief visit by an international palliative care expert, at which time an internal, project-specific palliative care protocol was created. In the intervening period, palliative care was increasingly discussed as an operational priority at the headquarters level. Project leadership explicitly identified palliative care as a strategic priority in the project’s 2021 Annual Plan, including the need for a “cultural approach to tackle ‘sensitive’ issues [such] as palliative care”, particularly for neonates [21]. During this time, the palliative care resources that MSF staff used most commonly were the MSF Neonatal and Pediatric Care Guidelines [31–33]; however, the sections devoted to palliative care are brief and focus on decisions to discontinue or forgo resuscitation, with some discussion of symptom relief. In April 2021, MSF-Spain published a comprehensive palliative care guideline [34] which is based on several international resources including the WHO guides for integrating palliative care into pediatrics and into humanitarian crises, the Global Atlas of Palliative Care and several others [1, 2, 35–39]. While there is a national guideline for palliative care in Bangladesh, the information related to pediatric and neonatal palliative care is limited [40].

The purpose of this study was to explore how MSF’s commitment to integrate palliative and end-of-life care relates to the values, priorities, and pressures experienced by MSF staff in a particular local context to inform the palliative care integration strategy at Goyalmara Hospital, within MSF, and in the humanitarian sector more generally. The research questions guiding this study were: 1) what are humanitarian healthcare providers’ moral experiences of facing and providing palliative and end-of-life care at Goyalmara Hospital, 2) how are healthcare providers’ moral experiences shaped by palliative care guidelines, training, mentoring, and palliative care integration strategies, and 3) how does gender, profession, experience, and position within MSF influence healthcare providers’ moral experience?

## Methods

### Methodological and theoretical orientation

This focused ethnographic study was guided by Hunt and Carnevale’s moral experiences framework [12] and Arthur Kleinman’s work on moral experience [41, 42]. Focused ethnography involves the classic strategies of traditional ethnography, including interviews, participant-observation, field notes, and document analysis with a shorter period of field work and focused research questions [43]. Moral experience is defined as, “a person’s sense that values that he or she deem important are being realised or thwarted in everyday life” [12], and is situated in social contexts that influence the meaning assigned to situations and interactions [12, 43].

### Setting and timeline

**Fig 1:**
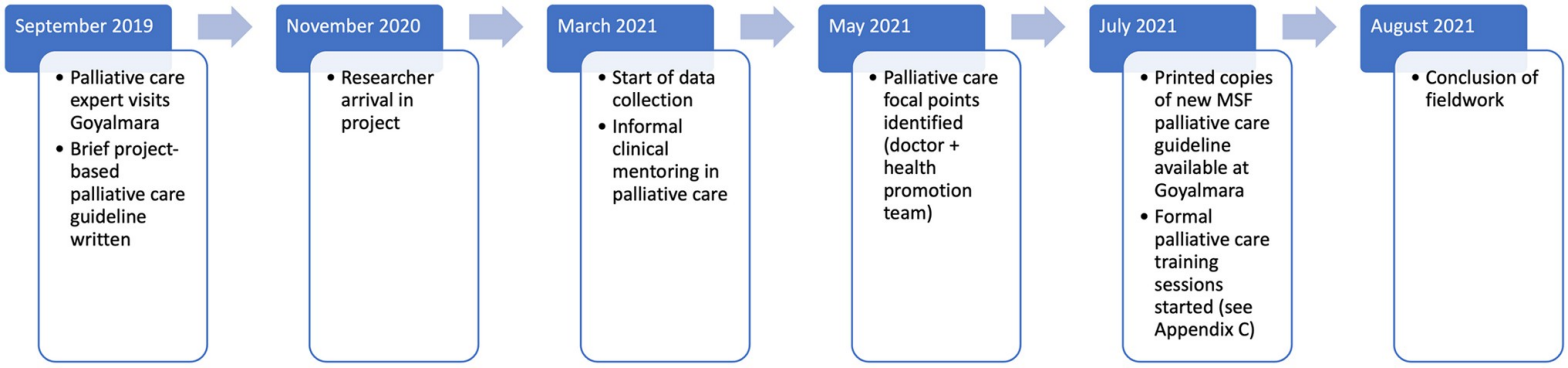
Palliative care program and research timeline.

This study was carried out at the Medécins Sans Frontières Goyalmara Mother-Child Hospital in Cox’s Bazar, Bangladesh, with formal data collection taking place between March-August 2021. Since one of the goals of this ethnography was to inform the development of a palliative care program, data collection took place alongside palliative care program implementation including creation of a formal palliative care pathway and training sessions in July and August 2021 (see S1 Appendix). A summary of the research and palliative care program timeline is found in Fig 1.

Goyalmara Hospital is located adjacent to the Rohingya refugee camps and serves patients from both the Rohingya and Bangladeshi host communities. The vast majority of clinical care is provided by staff hired in-country, known colloquially within MSF (and most international NGOs) as “national staff”, while “international staff” are usually on shorter term 3–12-month contracts and tend to occupy management or coordination level positions. The language of “national staff” and “international staff” is increasingly problematized within MSF; however, at the time of data collection this terminology was commonly used. While MSF has taken steps to address inequities around human resources practices and ensure that employment position is based on skills and experience rather than country of origin, ongoing challenges persist [44]. MSF strives to hire staff from within Cox’s Bazar district; however, due to an inadequate number of qualified professionals available locally, most doctors, nurses, and other professional staff at Goyalmara are from the capital city of Dhaka or other parts of Bangladesh. Bangladeshi staff from Cox’s Bazar district often speak Chittagonian, a Bangla dialect that is mutually comprehensible with Rohingya; however, staff from outside the district struggle to communicate with Rohingya families. Cultural, ethnic, and linguistic differences between international staff, Bangladeshi staff from outside the district, locally hired Bangladeshi staff and families, and Rohingya families impacted staff-patient relationships and the delivery of palliative care.

### Researcher positionality and conceptualization of palliative care

As lead researcher for this project, I (RY) am a pediatric critical care nurse with 16 years of nursing experience in the USA, Canada, and internationally with MSF. I am a PhD candidate with a master’s degree in public health and community health nursing and have been coordinating qualitative research studies over the past five years. During the period of fieldwork, I filled several roles at Goyalmara Hospital including neonatology nurse manager, clinical educator, palliative care program lead, and researcher. Throughout my time in the project, I was not involved in hiring or disciplinary functions as a manager, and once formal data collection began, I stepped back from my role as manager and did not have formal supervisory authority over study participants; however, as international staff, national staff continued to see me as holding a position of authority. My social location as a Canadian nurse of European ancestry may have impacted staff members’ willingness to share their perspectives openly; however, the rapport I developed with staff over the course of my 10 months in the project seemed to mitigate this. While not all staff may have felt comfortable being candid with me, there are many examples throughout the data set of staff professionally critiquing or contradicting international staff authority and directly challenging my choices related to the palliative care program, increasing the trustworthiness of the data and findings.

As an MSF international staff nurse, I occupied a unique position in that I could have been considered a participant in this study if data collection were performed by another researcher. My role in leading palliative care integration at Goyalmara towards the end of the study resulted in a complex interaction between my own views on palliative care, data collected as part of the study, and the emerging palliative care program content and priorities. My positionality and embeddedness in the study site inarguably influenced the findings; however, a researcher with no clinical training or involvement would come with their own set of biases that are different in content but not significance. Throughout the study design, data collection, analysis, and manuscript preparation, I consulted frequently with co-authors who offered important insider and outsider perspectives in relation to the Bangladeshi healthcare system, MSF, and the Rohingya refugee response. Reflexivity and the potential influence of my beliefs and assumptions about palliative care on staff moral experience were frequent topics of these analytic team discussions.

My views on palliative care are strongly influenced by the World Health Organization and other guidelines previously described. I understand palliative care to be holistic care aimed at relieving physical, psychological, social, and spiritual suffering for individuals and families facing a life-threatening illness and consider end of life care to be part of this broader approach. However, as a pediatric critical care nurse, my clinical experience related to palliative care is primarily in the area of end of life care. When I first arrived at Goyalmara, I was hoping to engage a broader definition of palliative care including care for children with longer-term palliative care needs but given the urgent need for end of life care services in the NICU and PICU, our inability at the time to offer home-based services, and the prevailing conceptualization of palliative care among staff, I found that my own model of palliative shifted in focus towards end of life care. An important dimension of my understanding of palliative care is the necessity of prioritizing patient and family preferences and values. While authentic shared decision-making remains a significant challenge in the Canadian healthcare system, I was coming from a context where, in my experience, most families expect to be engaged in these types of decisions. As a result, the lack of parental involvement in most aspects of palliative care decision-making at Goyalmara was quite noticeable when I first arrived. As time passed, I became habituated to this way of working and increasingly aware of the contextual factors including linguistic diversity and steep power differentials that made shared decision-making challenging, albeit no less important.

### Data collection

We collected four types of data as part of this study: field notes based on participant observation, individual semi-structured interviews, focus group discussions (FGDs), and palliative care related documents. Data were primarily collected by RY, with the support of MH who conducted Bangla-language interviews and FGDs. Prior to the start of data collection, RY and MH shared written and verbal information about the study with staff during clinical handover and in meetings with various professional groups including study aims and procedures, information regarding how staff could opt-out of observation-based data collection, and an invitation to participate in interviews and FGDs. Staff were given the option to choose to participate in an FGD or individual interview; and following the FGD, they could choose to take part in an individual interview if they felt they had more to share.

#### Participant observation

RY was actively engaged in the day-to-day activities at Goyalmara Hospital, so was able to observe a diversity of situations including clinical rounds, cardio-pulmonary resuscitation (CPR) events, conversations with families, training sessions, and meetings—during a variety of time periods (day, evening, and weekend shifts) and with various members of the healthcare team. RY took brief notes during the day and prepared more extensive field notes and reflexive memos in the evenings. The purpose of observation data was to contextualize other data sources, to triangulate verbally elicited data with staff behaviors and non-verbal expressions, to identify particularly salient cases to be discussed during individual interviews and FGDs, and to refine individual interview and FGD guides. Observations were focused on the context, staff experiences, and interactions rather than on patients and families.

#### Individual interviews

We carried out 22 semi-structured interviews with doctors (7), nurses (4), mental health counselors (3), health promotion staff (3), midwives (2), a health assistant (1), medical translator (1), and pharmacist (1) who were involved in providing palliative and end-of-life care to patients and their families. Bangladeshi and Rohingya health promotion (HP) staff play a critical role in providing health education, translation, and cultural mediation services at Goyalmara. A summary of participant characteristics can be found in Table 1. All staff who interacted with patients and families, or who were involved in the palliative care program were invited to participate in an individual interview (approximately 70 staff in total). Staff either approached RY directly to express interest in participating, or in some cases individuals with key roles in the palliative care program, those who were involved in potentially salient patient care scenarios, or those who had expressed strong views about palliative care were approached by RY directly to request an interview. We used maximum variation purposive sampling [45] to ensure adequate representation based on profession, nationality, gender, and diverse views on palliative care. Given the ethnographic methodology, we did not begin with a predetermined sample size. The final sample size was determined by the number of staff willing and able to participate given their busy clinical schedules, as well as the duration of RY’s contract in the field. We conducted interviews using a semi-structured interview guide that we developed based on the literature and moral experiences framework (see S2 Appendix). We iteratively modified the interview guide over time as our understanding of the context and issues salient to staff deepened. Participants were encouraged to explore related topics they felt were relevant, and clinical situations observed during fieldwork were often used to promote discussion.

**Table 1:** Interview and focus group discussion participant characteristics.

		Individual Interviews	Focus Group Discussions
**Total participants**		**22**	**18**
**Profession/role**			
	Health promotion staff	3	5
	Nurses	4	10
	Doctors	7	-
	Mental health staff	3	3
	Midwives	2	-
	Medical translator	1	-
	Health assistant	1	-
	Pharmacist	1	-
**Nationality/ethnicity**			
	Bangladeshi	15	18
	Rohingya	2	-
	African	1	-
	East Asian	2	-
	European	2	-
**Gender**			
	Men	10	8
	Women	12	10
**Language used during interview/FGD**			
	Bangla	5	16 (4 FGDs)
	Rohingya	1	-
	English	16	2 (1 FGD)

*Three participants took part in both an individual interview and FGD, therefore 37 unique individuals took part in interviews and FGDs.

Interviews were conducted in English, Bangla or Rohingya depending on participant preference. RY conducted all English language interviews as well as the Rohingya interview with simultaneous translation by a trained Rohingya translator. MH conducted Bangla interviews while providing periodic English summaries so that RY could ask follow-up questions. RY attended all interviews and took detailed notes during each interview. Interviews were audio-recorded, transcribed, and translated into English as needed. RY checked all English transcriptions for accuracy and anonymity, and a member of the research team (PH or MH) or a second transcriptionist checked all Bangla and Rohingya transcriptions, adding notes regarding cultural meaning and significance. Interviews were conducted in a private space either in the hospital or office depending on participant preference and lasted between 38 and 105 minutes (mean of 63 minutes).

#### Focus group discussions

Five focus group discussions involving a total of 18 participants were conducted with the health promotion team (5 participants), mental health team (3 participants), and nurses (3 FGDs with a total of 10 participants). A summary of participant characteristics can be found in Table 1. The purpose of this additional data collection method was to promote open dialogue among staff as we began integrating palliative care in the project, to explore how staff moral experiences varied by professional role, and to allow options for staff who felt more comfortable sharing in a group setting. FGDs were scheduled for each of the professional groups with a major role in providing palliative care, and RY shared an invitation with staff during clinical handovers and through departmental supervisors. An FGD was not conducted with doctors due to scheduling challenges; instead, individual interviews were more acceptable to this group. Nurses seemed to prefer the FGD format over individual interviews, so an FGD was arranged for nurses with women only (4 participants), men only (4 participants), and a mixed-gender group (2 participants). FGDs were conducted using a semi-structured interview guide (S3 Appendix) and were audio-recorded, translated, and transcribed in the same fashion as individual interviews. All FGDs were conducted in private spaces within the hospital and lasted between 65–95 minutes (mean of 78 minutes).

#### Institutional documents

We collected documents relevant to palliative and end-of-life care including MSF pediatric, neonatal, and palliative care clinical guidelines [31–34, 46], the project annual plan [21], monthly situation reports [30, 47–49], admission criteria and referral policies [50, 51], as well as palliative care training and programmatic documents that RY created towards the end of her assignment at Goyalmara [52–54]. All documents were written in English. International staff and national staff in supervisory level positions were aware of the project annual plan and participated in writing the monthly situation reports. The MSF pediatric and neonatal care guidelines [31, 33] were well-known by all staff and the brief sections pertaining to palliative care in these guidelines were referenced frequently by physicians and nurses. A more comprehensive set of palliative care guidelines [34] were published in April 2021 and a printed version became available in the project in July 2021. Towards the end of the period of fieldwork, staff were aware of these guidelines and beginning to use them in practice. Documents were reviewed by RY and MSF-affiliated coauthors. Since the focus of the study was staff moral experience, these documents were not primary data sources but instead were used to shed light on the context and how it impacted staff moral experiences [41].

### Data analysis

The analytic process began in the field so that emerging themes could be explored in ongoing data collection activities, and gaps in the ethnographic record identified [55]. Formal data analysis followed a modified version of the Qualitative Analysis Guide of Leuven (QUAGOL) [56, 57]. The QUAGOL approach aims to encourage researcher creativity and avoid overreliance on mechanical coding procedures by postponing and de-emphasizing the coding process [56]. Members of the research team recorded comments and reactions in the transcript margins during the data cleaning process and during repeated readings of transcripts (RY, MH, PH, LS, SB, JP) and field notes (RY). Based on these notes and analytic discussions with co-investigators, RY wrote narrative summaries of each interview and FGD transcript which aimed to document a cohesive story of each participant’s moral experience. Using the narrative summaries of the first 11 interviews and 1 FGD, RY inductively developed a list of concepts relevant to the research questions and created a preliminary codebook. As narrative summaries were completed for the remaining data and formal coding began, additional codes were added that were not adequately represented in the preliminary codebook.

Consistent with the ethnographic methodology [55], RY coded all field notes and transcripts in Nvivo qualitative data analysis software (Release 1.6.2). Then we used a variety of approaches including role ordered matrices [58] and hand-drawn diagrams to organize the isolated concepts and develop a “meaningful conceptual framework or story-line” [56]. Constant comparison between narrative summaries, coded data, and emerging themes was used to explore and test the findings [56]. Throughout the analytic process, RY met with Bangladeshi as well as both MSF and non-MSF co-investigators to discuss emerging findings and ensure that interpretations incorporated both insider and outsider perspectives. Since none of the study participants were first-language English speakers, we have made minor grammatical corrections to certain quotations when necessary to improve clarity while aiming to retain the way that MSF staff from diverse countries and with varied levels of English proficiency communicate. Given the complexity of the study context and the ethnographic approach, data saturation was not a relevant or feasible goal; however, towards the end of the period of fieldwork we saw significant repetition as well as variation within the collected data and felt that an adequate depth of understanding of the context had been achieved. In August 2021 we held an in-person discussion session with Goyalmara staff to gather feedback on early findings, and in November 2022 we arranged a similar online restitution of findings event to obtain feedback on the results presented here.

### Ethics

Ethical approval for this study was obtained from the Bangladesh University of Health Sciences Ethics Review Committee (ID: BUHS/ERC/EA/21/31) and the MSF Ethics Review Board (ID: 20109). Staff were offered the option to decline inclusion of any information about them in field notes by contacting RY, MH, their direct supervisor, or the medical coordinator. During individual informal conversations, we confirmed that staff agreed that a summary of the conversation could be included in field notes. All individual interview and focus group discussion participants gave prior written informed consent. Additional information regarding the ethical, cultural, and scientific considerations specific to inclusivity in global research is included in the S1 Checklist.

## Results

A major theme that emerged from our analysis and the focus of this paper was the centrality of decision-making to MSF staff’s moral experiences of providing palliative care, and the way that conceptualizations of palliative care, moral and religious values, and various forms of authority shaped their experiences of palliative care decision-making. The decisions that were most salient to staff included decisions to transition to comfort-focused care at end of life, use of life-prolonging medical treatments and cardio-pulmonary resuscitation, as well as referral to higher level of care in a resource-constrained context. While some of these issues could be considered peripheral to palliative care, they were the issues that MSF staff grappled with in the specific local context of Goyalmara Hospital.

### ‘Putting patients in palliative care’: Staff conceptualizations of palliative care

To understand the staff’s moral experience of providing palliative and end-of-life care, it is necessary to first understand the meaning of these concepts in the context. The model of palliative care being practiced during the period of fieldwork at Goyalmara Hospital was in transition and characterized by significant conceptual ambiguity. The prevailing understanding of palliative care among national and international staff was care that prioritized comfort for infants and children who were not expected to survive; when we had reached the limit of available medical treatment and “there was nothing left to do” (FGD-01, Mental Health Team). “Counseling”, which staff understood to mean both sharing difficult news and information with parents, as well as demonstrations of empathy and psycho-social support, was central to how staff understood palliative care. The language that MSF staff used to describe palliative care may be more consistent with international definitions of end of life care, hospice, or care of the dying patient [2, 35, 37], yet it was the English term ‘palliative care’ that staff used in practice so that in the terminology we will use in this paper.

Staff felt that palliative care was needed for extremely low birth weight and/or premature neonates, neonates with severe perinatal asphyxia and congenital malformations, as well as children suffering from congenital heart disease, leukemia, liver and renal failure, thalassemia, and infectious diseases such as meningitis that were not responding to treatment. They were aware that some children for whom palliative care was the focus may have survived if they had access to a higher level of care, and children needing palliative care were often described as having an incurable illness “in our context” (Doctor-03), particularly by international staff and Bangladeshi doctors. Few staff made a distinction between palliative care and end-of-life care other than an international staff doctor and nurse, and those Bangladeshi doctors who had taken part in a recent formal palliative care training. These Bangladeshi doctors and international staff felt that palliative care could be offered even if the child was expected to survive for weeks or months if definitive treatment to cure their underlying condition was not available.

Many non-physician staff such as health assistants, translators, health promotion and mental health staff and to a lesser extent nurses, had a very narrow understanding of when palliative care was indicated and believed palliative care was what happened after unsuccessful cardio-pulmonary resuscitation (CPR). A medical translator, health assistant and midwife who took part in individual interviews understood palliative care to be nearly synonymous with critical illness and believed it involved ongoing efforts to save or extend life, even while they acknowledged that the likelihood of survival was low. Importantly, these staff had not received any formal training related to palliative care at the time of their interviews, and the health assistant and translator felt that they were often neglected when it came to formal training which tended to focus on doctors and nurses.

Staff frequently used language such as “putting a baby in palliative care” (Doctor-02) or “entering” a patient in palliative care (International Staff-01) to mark the transition between disease-directed life-saving care, and comfort-focused palliative care. Putting a patient in palliative care often meant discontinuation of life-prolonging treatments such as CPR, intravenous (IV) fluids, and oxygen. There were staff who felt that certain life-saving or prolonging therapies should continue when a patient was in palliative care, but even in these cases, they saw palliative care as having clear boundaries: children were either in palliative care or not. Staff occasionally described patients as labeled or “marked as an end-of-life care patient” (FGD-03, Nurses), implying a certain degree of stigmatization, and several participants including doctors, nurses, mental health, and health promotion staff were concerned that use of the palliative care label could lead to inappropriate discontinuation of treatments, inadequate monitoring, or even neglect. Towards the end of the period of fieldwork, there was a growing recognition among staff that palliative care interventions should be individualized based on the patient’s condition and family preferences, as explained by a Bangladeshi doctor who said, “previously I had a thought that palliative care means no antibiotics, no IV fluid, nothing, only the supportive care, but here I realized that it varies patient to patient condition” (Doctor-04).

Staff from a variety of professional backgrounds emphasized the importance of not abandoning palliative care patients and families, including a Bangladeshi doctor who stressed that these children should not be skipped during medical rounds (Doctor-01), a medical translator who said, “there should be no negligence in the treatment of such children” (Translator-01) and a health assistant who felt they should be “monitored very closely” (Health Assistant-01). An international doctor felt that it was critical to ensure staff understood what palliative care was, “not to misuse it” and to “select appropriately the kind of patient that will benefit and will enter the palliative care” (International Staff-01).

### Doing our best and doing no harm: Staff moral and religious perspectives on palliative care decision-making

Given the implications of transitioning to palliative care and the bounded nature of the concept, decisions to begin palliative care at Goyalmara were emotionally and morally complex. Decisions to forgo or continue life-prolonging and invasive medical treatments were the most contested aspects of palliative care at Goyalmara. Staff members’ views were informed to varying degrees by their sense of moral obligation to do their best and act on behalf of their patients, a sense of obligation to do no harm, and for most national staff, spiritual beliefs regarding God’s role in saving or extending life. The international staff and Bangladeshi doctors we interviewed often prioritized the obligation of healthcare providers not to cause harm through the continuation of futile and burdensome medical treatments. As one international pediatrician explained, “we need to accept that as health providers, we, the first thing we always need to remember is to not harm, do not harm. And by giving treatments, many, many times we are harming. So, we need to step back a little and think about which treatments are we giving, why, what is our goal, and what are the side effects” (International Staff-05). Many national staff emphasized their moral obligation to do their best on behalf of their patients, with phrases such as “we have tried our best” (FGD-01, Mental Health Team) present in nearly every focus group and interview. While some staff believed that doing our best could include palliative care interventions to relieve pain and attend to the families’ emotional and spiritual needs, for many national staff, doing our best meant doing everything possible to save and extend life. Their opinions varied, but national staff often expressed a sense of obligation to continue IV fluids, oxygen, antibiotics, and enteral feeding. Nevertheless, among national staff nurses and doctors, the sense of obligation to continue trying was tempered by their awareness of the discomforts and burden of these medical treatments, and doctors and nurses were more likely than other national staff to accept or even advocate for discontinuation of burdensome interventions for patients receiving palliative care.

While continuation or discontinuation of IV fluids, oxygen and enteral feeding was fairly contested, staff often understood CPR to be futile, painful for the child, traumatic for families and, if poorly understood in the community, potentially damaging to MSF’s reputation. Many staff accepted or even pushed for avoidance of CPR when it was likely futile; however, some nurses felt that it was important to provide CPR “out of a responsibility, it has to be given to maintain formalities or protocols” (FGD-03, Nurses) or because they believed there was a chance of survival. Other nurses experienced a tension between hopefulness that CPR could save the child’s life and their own observations of post-CPR outcomes at Goyalmara. As one nurse explained: “it is better to not give CPR, then again it feels like maybe the child could improve; however, I know it will not happen but there is a confusion in my mind” (FGD-04, Nurses). At Goyalmara, CPR was limited to chest compressions and non-invasive (bag-mask) ventilation since there was no capacity to offer endotracheal intubation, and post-resuscitation medical interventions such as infusions to support blood pressure and dialysis were not available. The confusion that staff experienced surrounding the utility of CPR was likely influenced by years of training they had received focused on the mechanics and importance of CPR, which conflicted with their own observations of post-CPR outcomes in practice. International staff who led these staff trainings over the years were often relatively junior with little exposure to pediatric death or in-hospital arrest without access to advanced critical care support and may have lacked the clinical experience to recognize when CPR was futile in this context.

For national staff, spiritual beliefs about how God intervenes in the world were strongly connected with their sense of moral obligation to do their best and their beliefs about continuing or forgoing life-prolonging treatments. While religion and spirituality were not the original focus of interview and focus group discussion guides, these concepts came up frequently and spontaneously in discussions with national staff and were key to understanding their moral experiences of palliative care. Most national staff at Goyalmara were Muslim, with a smaller number of Buddhist, Hindu, and Christian staff. International staff who took part in interviews either described themselves as non-religious, or religious themes were not discussed in their interviews.

The national staff’s core belief about God’s role is best summarized by a nurse who said, “now above all we have the belief, those of us who are Muslims, that we are just a medium. Birth and death are controlled by the One” (FGD-04, Nurses). When a child died despite their best efforts, national staff often understood this to mean that it was not God’s plan that the child should survive. As a mental health counselor explained, “therefore, after giving as many times as it requires according to their ability, the rest has to be left to Allah” (FGD-01, Mental Health Team). These staff continued to believe that a miracle was possible but felt that further medical intervention would not affect the likelihood of a miracle and would only increase the child and family’s suffering. They seemed to connect palliative care with a sense of humility and acceptance of the limitations of biomedicine. As this nurse explained, “I cannot do anything, I have limitations, but I don’t have any right to burden sufferings, burden her emotional sufferings. Almighty is the best planner. I believe that Allah knows the best what will happen, so in many cases when I know I don’t have anything to do, I believe we should discharge the patient…I am accepting my limitation that I have nothing to do for your baby” (FGD-05, Nurses). For these staff, the nature of God’s role in determining the child’s fate meant that it was futile or even harmful to continue aggressive life-prolonging treatment once the team agreed that they had reached the end of what biomedicine could offer.

In contrast, other national staff felt that since the child’s outcome was in God’s hands, it was inappropriate for medical staff to make definitive predictions about survival. A mental health team member explained that some families will be resistant to discontinuing life-prolonging therapies because “you are not God that you can say that this is finished” (Mental Health Team-01). For some national staff, being a medium meant that their life-saving efforts left open the possibility that God may intervene on behalf of the child. Their belief that God was ultimately in control did not abdicate them of their responsibility to continue trying. One nurse expressed frustration with Rohingya caregivers who refused what he believed to be life-saving medical treatment saying, “they don’t understand, they think God will save them” (Nurse-02). He contrasts this with his belief that God will not save the child if you “do not [16] anything” (Nurse-02).

For many staff, being able to reassure themselves that they had done their best alleviated feelings of guilt and grief after the death of a child. As a Bangladeshi nurse explained, “if we can give [59], it feels good that we are trying for him. The result is either good or bad it does not matter” (FGD-03, Nurses). Another nurse said, “we explain to ourselves that we have given as much service as we can, it is in the hands of Allah whether he will survive or not” (Nurse-03). For other nurses, heroic efforts were not reassuring and left them with a sense of failure: “Even after running our procedure, the child did not survive. Then it feels bad that I tried, but it did not work” (FGD-03, Nurses). Staff felt that it was extremely important for families to understand that the medical team had done their best, both for the family’s emotional coping and to avoid incidents of blame directed at the team. While most national staff simultaneously believed that they had an obligation to do everything in their power to save the child’s life and that God was ultimately in control of the child’s outcome; for many staff, these beliefs were in tension. The diverse ways that these beliefs and obligations were interpreted impacted how staff felt about decisions to discontinue life-prolonging interventions for children receiving palliative care.

Based on data collected early in the period of fieldwork, we recognized that the prevailing conceptualization of palliative care was in tension with many of the staff’s sense of obligation to try their best. During palliative care training sessions and clinical mentoring that took place in July and August 2021, we emphasized the active nature of palliative care in assessing and treating symptoms, providing psycho-social and spiritual support, as well as the potential for interventions focused on reducing suffering to be offered alongside disease directed treatment in certain cases. There were participants who saw palliative care as highly compatible with action and effort on behalf of our patients and this conceptualization slowly grew and evolved during the period of fieldwork. One Bangladeshi doctor (Doctor-01) felt that particularly for patients with diagnoses like congenital heart disease or leukemia who were expected to survive for several weeks, months or even longer, continuing certain disease directed treatments may be appropriate. A mental health counselor believed that continuing to offer supportive care for a child with suspected rabies was important to ensuring the family did not feel abandoned. The counselor explained, “what seems admirable is that we gave them space at that moment, that we put them in palliative care. We did not tell them at that moment that, ‘it’s over, now you can go, or you may leave if you want to, we have nothing to do now’. We gave them care at that moment, it seemed very admirable to me (FGD-01, Mental Health Team).

Active treatment of pain and breathlessness using opioids at end of life was new for most staff and access was unreliable; however, several staff described positive experiences after the hospital began introducing these medications. One Bangladeshi doctor described her first experience using morphine at end of life in this way, “the death was so painless. The end-of-life care went very smoothly. Baby was not irritated. Baby was absolutely calm and quiet… So that moment I found, this end-of-life care helps, because that moment mother and father was very calm. They said, okay my baby will die, it was a peaceful death. What else we could have asked from God?” (Doctor-02). An international doctor explained why shifting the perspective so that palliative care was understood as active care was so critical both to staff acceptance and her own coping with repeated losses:

I think that what helps to cope is to focus on the positive points that you can make, the difference that you can make to the family…because you will see that some families they appreciate a lot that you don’t give up or that you still are there and especially because we can make a difference in their symptoms treatment. So I think that’s the, to focus on the positive things that we can do, that they are a lot, and to try to be in the place of the family

(International Staff-01).

### Power, authority, and palliative care decision-making

There were four main sources of authority that informed decisions to “put patients in palliative care”, to continue or forgo certain medical treatments, and staff’s moral experiences of those decisions: family authority, medical authority, international staff authority, and the authority of protocols, guidelines, and policies.

#### Family authority

The new MSF-Spain Palliative Care Guidelines, introduced in the project in July 2021, describe the importance of involving families in care-planning decisions [34]; however in practice, family engagement in palliative care decision-making was usually limited to families accepting or refusing the plan of care presented by the medical team. Given the lack of familiarity with a shared decision-making model among both staff and families, as well as large power differences and language barriers, staff struggled to engage in authentic discussions about patient and family preferences. According to nurses who took part in two focus group discussions (FGD-03 and FGD-04), Bangladeshi families tended to be less accepting when staff suggested a transition to palliative care and often requested referral even when a higher level of care was not available. In contrast, one nurse explained that Rohingya families usually accept what the team suggests and that “they say in their language that you do what you have to do, as much as you have to do” (FGD-03, nurses). While Bangladeshi families were seen as more likely to negotiate with the team regarding the child’s plan of care, Rohingya families usually asserted their authority through their decisions to remain in hospital or take their child home.

At the time of this study, the team did not have the capacity to offer home-based palliative care and patients often did not reside in the camps where our partners (HMBD or IOM) were able to offer follow-up. Therefore, particularly for patients nearing end of life who were experiencing distressing symptoms, staff found it upsetting when families chose to take their child home and the mental health and health promotion teams spent a significant amount of time trying to “convince” families to remain in hospital. As a member of the health promotion team explained, "another thing we can explain to them is that if you see your child dying without treatment when he is in his last stages, his suffering will increase. In that case, many times, we keep them for a longer time by convincing them, and then they understand things and wait until the last stage” (FGD-02, Health Promotion Team).

Despite the limited family engagement in palliative care decision-making, several national and international staff advocated for greater family involvement, including a Bangladeshi doctor who said, “we can explain but the main decision should be taken from the patient or patient attendants. That’s their patient that’s going to die” (Doctor-05). In May 2021, a doctor and health promotion team member were identified to improve communication with patients and families receiving palliative care. The health promotion team focal point played an important role in assessing families’ preferences, arranging transfer to a private room, and facilitating visits by religious and traditional healers if desired by the family.

#### Medical authority

Decisions surrounding palliative care and resuscitation were primarily understood to be medical decisions at Goyalmara. Many staff understood teamwork and interdisciplinary decision-making to be an important or even defining feature of palliative care, yet the doctor was understood to be the leader of the interdisciplinary team. As one nurse explained, “the doctor is the team leader. They make decisions after discussion” (FGD-03, Nurses). Even if doctors were the ultimate decision-makers, nurses saw themselves as having an important role in palliative care-related decision-making: “because I am a nurse, I have observation, I have a different field, and from my field I have something to give decision. I have some opinion to express” (FGD-05, Nurses). When it came to making decisions about CPR, some nurses felt that they were included in the conversation; however, others saw themselves as merely acting on decisions made by the medical team. During a focus group discussion, a nurse described a particularly upsetting situation:

There was a baby, for a long time here. 30-minute ventilation period was over, at that time the doctor said that the child will get CPR. I was seeing that nothing would happen even with CPR. Since we follow the doctor’s orders, I have to perform the CPR in that case as well. Many such incidents happen, brother, at that time it feels bad that nothing will happen even after performing CPR. Still I have to perform CPR, since I am a nurse and I have to obey the orders of the doctors. It feels so bad at that time that the baby is dying, there is nothing left, yet we are continuing CPR

(FGD-04, Nurses).

Another nurse described how these experiences of feeling compelled to follow doctors’ orders have led her to question her decision to become a nurse: “we feel bad, and then of course I feel that why did I become a nurse, not a doctor?” (FGD-04, Nurses).

Members of the health promotion and mental health teams, as well as medical translators and health assistants generally did not see themselves as having a role in making decisions about palliative care but instead saw their role as explaining the medical team’s decision to the family. During interviews and focus groups, staff who were not doctors or nurses frequently added the caveat, “doctors know better than us” (Health Promotion Team-01) or “I am not a medical person” (Health Promotion Team-03) when they expressed opinions about the medical aspects of palliative care. While these participants were often referring to their relative lack of medical training—which they believed meant they had no role in palliative care decision-making—their comments also pointed to a lack of confidence in the validity of their opinions. However, there were others who recognized their potential contributions to interdisciplinary team meetings including sharing information about their observations and discussions with families, as well as cultural and contextual knowledge. One mental health counselor acknowledged that it was valuable for the mental health and health promotion teams to take part in care planning discussions saying, “if the HP team knows something they share, everybody shares what they know and then everyone gets to know about the patient… I think my role is to get knowledge about the patient, because I have to counsel that patient, the patient caretaker” (Mental Health Team-02).

As we progressively placed greater emphasis on interdisciplinary team decision-making, tensions occasionally surfaced around the role and degree of authority that non-physician team members should have regarding palliative care related decisions. The medical team sometimes found it frustrating or burdensome when they had to wait until the various team members could be assembled. As one international doctor expressed,

I like the idea of being a multi-disciplinary team and everything, and I’m not changing it, but I think from my point of view that the conversation should be taken first, only with the people involved, like all the doctors in the ward and all the nurses in the ward at that moment and well, health assistants just for them to know…I cannot wait for the other ones to come, they never have anything to do of course because they are not health staff, like direct health staff

(International Staff-05).

When nurses, health assistants, translators, mental health, and health promotion staff were present for care-planning discussions, they rarely contributed to the conversation; often simply nodding when asked if they agreed with the plan. While power dynamics and deference to authority certainly impacted staff engagement, it was clear that a lack of training and clarity surrounding the role of non-physician staff when it came to palliative care decisions also contributed to their lack of involvement. As one nurse clearly articulated, “as a palliative care nurse, I have to learn first, then I can give my opinion: the team should do this, team should not do this… But first of all I have to learn, because if I have a lack of knowledge then I cannot participate in decision-making” (FGD-05, Nurses). Certain decisions, such as prescribing or withholding medical treatments, are ultimately medical decisions so in the absence of guidance about how they could contribute, non-physician staff struggled to understand their role.

#### International staff authority

While non-physician staff tended to see palliative care decision-making as led by the medical team, Bangladeshi doctors and some nurses emphasized the role of the international staff, particularly international pediatricians, in palliative care related decision-making. Since most national staff doctors did not have specialized pediatric training when they began working at Goyalmara, international pediatricians were hired to support the team. The authority of international staff at Goyalmara was connected to their positions as managers in the human resources hierarchy, their specialized expertise in pediatrics, as well as the pervasive deference to foreigners that is a legacy of British colonialism in Bangladesh [60].

The two international pediatricians who took part in interviews experienced varying degrees of acceptance or discomfort with their perceived power. One pediatrician felt that it was not their role to be excessively authoritative, saying that in “some cases you want to be flexible and not like being the captain of the boat, only giving orders” (International Staff-01). They felt that it was important to make decisions as a team but that in cases when the rest of the team wanted to provide care that they believed was harmful to the patient, they felt they needed to insist because, “it’s not like a democracy” (International Staff-01). Another pediatrician felt alone in carrying the weight of particularly ethically complex decisions. They described involving the team, but “at the end you know, I am taking this kind of decision, so it’s all on me … the staff keep looking at me like this, ‘what do you want to do? … and I understand eh? I don’t want them to take this kind of decision and feel like they are killing a baby. I prefer that they put that on me, they don’t take that to home” (International Staff-05). As pediatric specialist and most senior clinician, they seemed to feel a sense of responsibility to protect the national staff doctors; however, even if well-intentioned, this left the pediatrician bearing that moral weight alone and assumes the Bangladeshi doctors wished to be protected in this way. The pediatrician believed that relationships with advisors and reassurance of judgment-free lines of communication would have increased their confidence to seek support in the case of ethically complex clinical situations.

While some Bangladeshi doctors described feeling supported by international doctors as they made decisions, others experienced distress when decisions were made that contradicted their values. One Bangladeshi doctor described feeling that she could speak up: “I think we should speak our mind and we need to listen to our mind. If the pediatrician tells us no, it’s palliative care, if we are not agree with her decision we can discuss… but it’s not like that, that the pediatrician told so and we need to do so” (Doctor-04). When describing his concerns about decisions made for a particular palliative care patient, another Bangladeshi doctor alluded to the presence of disagreement among staff who felt they could not speak up: “and not only me, I know the other doctors and other staff also, but they can’t express. But I express, I always tell my opinion” (Doctor-05). He believed that in Bangladesh it was uncommon for healthcare staff to question their superiors for fear of job loss or lack of career advancement, and this was why other staff did not express their concerns. For non-physician staff, particularly those hired from among the host community, he said that “MSF here has given them the best job opportunity than the other places. So, they’re very careful about their job” (Doctor-05).

#### Authority of protocols, guidelines, and policies

The authority of protocols, guidelines, and policies at Goyalmara Hospital was rooted in the staff’s understanding that they were based on scientific evidence and expert knowledge, that MSF staff were expected followed them as the standard of care in MSF projects, and that they promoted consistent quality of care across MSF contexts and countries. For many staff, both national and international, the introduction of palliative care guidelines was met with enthusiasm; however, the staff’s relationship to guidelines was also shaped by the authority and impermanent presence of international staff in the project. In most cases, international staff held management positions and were in the project for between three months to one year. The impact of these transitions on palliative care decision-making was most clearly described by a Bangladeshi doctor who explained that prior to the introduction of the new palliative care guidelines,

Every person has their own thinking so maybe some pediatrician is telling us that no, in palliative care we will do no CPR and no ambu bag but we will continue the IV lines, all the medications up to the patient’s death and some pediatrician is telling us that no, we will not do anything. So there is no protocols for this palliative care, so we don’t know actually what we are doing and what we are not doing and we are listening that time only the person to person who varies this palliative care thinking

(Doctor-03).

She expressed significant frustration with the inconsistent mentoring she was given by various international doctors about palliative care and felt that a guideline would support her desire for more consistency and clarity around palliative care decision-making. During a focus group discussion, two Bangladeshi nurses described a similar experience of international staff impermanence:

Male Nurse: When new expert comes, they introduce new laws, new rules, new protocols. When she left, new one comes, she changes it. Our sisters, the caregivers, nurses, they become disoriented. Now what to do, old expert gone, new has come.

Interviewer: That must be very frustrating, is it frustrating for you?

Female Nurse: No.

Male Nurse: No, not frustrating-

Female Nurse: No frustrating but, we are happy but we have become accustomed to it.

Male Nurse: Sometimes we feel uneasy but within a few days we are habituated with the new system

(FGD-05, Nurses).

The two nurses were careful to avoid direct criticism; however, they clearly experienced some level of ‘unease’ when successive international staff changed practice. The male nurse later requested that, “you should teach us about your protocols and standard techniques…if I know it clearly then before following your advice blindly, I will assess it. Is it true? Is it right or wrong?” (FGD-05, Nurses). He and other national staff seemed to suggest that protocols could be an empowering tool that national staff used in conversations with international staff to defend their point of view. International staff also saw clinical guidelines as an antidote to international staff impermanence because “as much as you stick to this [the guideline], it’s the thing that is going to remain” (International Staff-01).

Palliative care guidelines and other policies were also contested and challenged by staff. A Bangladeshi doctor (Doctor-05) and mental health practitioner (Mental Health Team-01) believed that while palliative care guidelines had value, we needed to consider the patient’s individual condition, patient or family’s preferences, and tailor the guideline to the context because “the patient, they don’t understand the SOP [standard operating procedure]. They understand their condition” [Mental Health Team-01]. Other national staff expressed significant frustration with referral policies that specifically excluded referrals for cancer and major congenital heart defects [50]. During an interview, a Bangladeshi nurse questioned why MSF did not pursue surgery for an infant who died of congenital heart disease asking, “why MSF did not do that? Because they [MSF] have a lot of money” (Nurse-02).

A Bangladeshi doctor described how deference to international staff authority and rigid loyalty to protocols, guidelines, and policies may serve to silence national staff because “they will not show courage to say against supervisor as MSF said to maintain the protocol, then no one will have courage to say their opinion” (Doctor-05). The authority of international staff was intertwined with that of protocols and guidelines so that international staff seemed to be the embodiment of the guidelines, and at times MSF institutional structures, in the eyes of the national staff. When asked how we could create true interdisciplinary palliative care teams where all staff felt comfortable expressing their opinions, a Bangladeshi doctor explained that unless national staff are given permission to question protocols, they are unlikely to express their honest opinion to international staff. His recommendation was:

We can tell them that, here we are a team, we are working here as a team, no one is better than, no one is smaller than you. We always give our opinion. Here is our protocol, it is made internationally after this observation but here we are going to apply this in your country…what is your opinion?

(Doctor-05)

## Discussion

### Summary of main findings

The main findings emerging from our analysis were related to the importance of staff conceptualizations of palliative care, moral and religious values, and various forms of authority to staff moral experiences of palliative care decision-making. Here we will discuss several key findings and related literature that are of relevance to the humanitarian sector as it moves towards integration of palliative care. First, we explore the centrality of spiritual beliefs and values to the national staff’s understanding and experiences of palliative care, and their interaction with the primarily secular values of humanitarian organizations. Secondly, we explore the implications of a passive framing of palliative care given staff’s sense of moral obligation to act on behalf of their patients, and the unjust access to medical care that characterizes humanitarian contexts. Thirdly, we discuss the importance and challenges of shared decision-making in humanitarian contexts and barriers that prevent national staff, non-physician staff, and families from expressing their views. Finally, we explore the protocolization of palliative care in humanitarian contexts and potential risks or limits of this approach.

### God’s will and doing our best

Despite MSF’s official secular positioning [61], Islamic ethics and spirituality were important “horizons of significance” [12] that shaped the national staff’s moral experience of palliative care. Although staff did not specifically mention these terms in their interviews, their beliefs about God’s will resonate strongly with the Islamic virtues of *tawakkul* (reliance on God), *sabr* (forbearance or steadfastness when facing hardship) [62], and *rida* (pious contentment in the face of difficulty) [63]. In interviews and FGDs, MSF nurses described themselves as “just a medium” (NUR-01, FGD-04, nurses), a role that implies a lack of control over the outcome, yet simultaneously seems to require action on their part.

The Islamic call to submit to God’s will has often been conflated with passivity and fatalism by Western and secular outsiders, and within Islam, the charge of fatalism has at times been framed as a negative trait [62]. Several ethnographies in Muslim contexts offer important re-framings of Islamic virtues such as *tawakkul*, *rida*, *sabr*, and the role of a “medium”. In Basit Kareem Iqbal’s ethnography of Islamic humanitarianism in Jordan, the leader of an Islamic NGO asserts that we must “both act and have trusting reliance on God (*tawakkul)”* [64]. The leader justifies this stance by citing a well-known hadith in which a man asked the Prophet if he should tie his camel to prevent it from wandering away or leave it untied and trust in God’s provision. In response, the Prophet exhorted him to “tie it and trust God” [64]. Similarly Saba Mahmood described how her pious Muslim study participants in Egypt believed that “fate does not absolve humans from responsibility” [65]. Two ethnographies conducted in Egypt explore the connection between the role of a medium and human action. In her ethnography among dialysis patients in Egypt, Sherine Hamdy quoted a terminally ill man who said, “you might think doctors can help- but if they can heal, they are only instruments of God’s unique healing abilities” [62]. And yet, Amira Mittermaier found that for devout Muslims in Cairo who spent their time preparing and distributing food for the poor, being a medium was far from passive and required daily effort to serve others [63]. These dialectical interpretations, where reliance on and submission to God are emphasized alongside a human duty to act, are consistent with the ways that many MSF staff interpreted these virtues.

The various ways that MSF staff interpreted Islamic virtues, their role as God’s medium, and their duty to act on behalf of their patients demonstrates the diversity of what Mittermaier refers to as “grammars of Islam” [63]. Hamdy found that among terminally-ill dialysis patients, *tawakkul* and *sabr* were interpreted and cultivated in different ways depending on the context and their assessment of the benefits and burdens of available medical interventions [62]. It cannot be assumed that the way that staff interpreted religious virtues in Bangladesh is transferrable to humanitarian interventions in other Muslim contexts, yet these diverse interpretations have implications for how humanitarian staff make and experience palliative care related decisions. One challenge that has been identified in the literature to integrating palliative care in humanitarian contexts is the cultural and religious "situatedness of dying” [6, 66]. At first glance, the staff at Goyalmara seemed to express vastly different values related to palliative care and the appropriateness of certain life-sustaining medical interventions for children receiving palliative care. Without minimizing important differences in staff’s belief systems, it is important to note that their underlying values including a sense of obligation to act on behalf of their patients, to not cause harm, and their recognition of the limited ability of healthcare workers to control or predict the future were quite consistent across both national and international staff. Perhaps one way forward is to explore those values that unify the team, finding common ground around which interventions are accepted and perceived to improve care by staff, families, and communities. This may lessen the potential for mistrust and moral distress, and improve acceptance of palliative care among staff, families, and communities, allowing for gradual integration of other palliative approaches over time. Without negating MSF and other humanitarian organization’s commitment to religious impartiality and neutrality [3, 61, 67], we would second Mittermaier’s call for humanitarian organizations to take seriously the spiritual and religious dimensions of the contexts where they work, particularly as we place greater emphasis on palliative care [63].

### Palliative care as active care

The findings of this ethnography highlight both the challenge and importance of addressing conceptual ambiguity and the passive framing of palliative care before implementing a palliative care program in a humanitarian context. Many of the tensions that MSF staff experienced were related to how palliative care was conceptualized as passive, implying the discontinuation of lifesaving or life prolonging medical care, which some staff felt was incongruent with their professional duty and sense of moral obligation to do their best on behalf of their patients. Given the resource constraints and complex prioritization decisions that occur in humanitarian interventions [6, 61], our findings raise the concern that if palliative care is understood in passive terms there is a risk that staff may not prioritize the needs of children perceived to be “hopeless” (Midwife-01), that they may see palliative care as an option to reduce staff workload, or fail to advocate for better access to life-saving care where appropriate.

Palliative care, as understood by large international health organizations and palliative care specialists, is highly consistent with MSF’s humanitarian mandate and MSF staff’s perceived moral obligations as it involves providing active treatment to relieve suffering alongside disease-directed treatment if appropriate [1–3, 68, 69]. The discrepancy between these formally published definitions of palliative care and local conceptualizations of palliative care, which were framed in more passive terms, is understandable given that palliative care is relatively new in Bangladesh and generally not included in undergraduate medical and nursing curricula [70, 71]. Our findings are consistent with a 2020 survey of Bangladeshi physicians which found that participants had good understanding of general palliative care principles, but believed that palliative care was only relevant for patients nearing end of life and that it discourages patients from pursuing curative treatments [70]. Based on this finding, we emphasized the active role of palliative care in relieving suffering during palliative care trainings and explored circumstances when palliative care could be offered alongside potentially life-saving interventions.

Palliative care in humanitarian contexts must continue to uphold the humanitarian ideal of action in response to human suffering [61, 67]. This may mean shifting focus from lifesaving to comfort-focused care for the patient in front of us when access to higher level care does not exist or is not available in the context due to global inequity in access to medical treatment. However, we must be cautious that palliative care does not relieve us of our humanitarian duty to bear witness and advocate for justice in access to life-saving care [3]. Efforts to scale up palliative care in humanitarian contexts must be accompanied by efforts to improve access to life-saving care at the project, country, organizational, and global level [6, 72, 73]. We stress the importance of re-evaluating admission and referral criteria prior to or alongside palliative care program integration to ensure that staff, patients, and families feel confident that teams are doing everything possible for their patients. While many valuable palliative care interventions are not resource-intensive but are the “small things” that staff can do for patients such as ensuring they are not alone [66, 74], integration of palliative care does require some degree of resource investment. The passive framing of palliative care and associated sense of powerlessness among some MSF staff was exacerbated by sporadic opioid access and lack of home-based care. Therefore, even though humanitarian contexts may limit access to certain palliative care interventions, it is critical that humanitarian staff have access to the tools needed to relieve their patients’ suffering to ensure that the message and reality of palliative care as active care are consistent.

### Power and shared decision-making

An important, if unsurprising, set of findings of this study was the way in which the authority of international staff informed palliative care related decision-making and the barriers to shared decision-making staff encountered both within the team and even more so, with patients and families. The pervasive hierarchies that exist in many contexts between patients and healthcare staff are exacerbated in humanitarian disasters [75]. One study which explored power dynamics between doctors and bereaved patients in post-tsunami Indonesia, found that patients used silence as a mode of resistance when they perceived that doctors were unwilling to listen to their concerns [75]. It is possible that Rohingya families were similarly using silence when they chose to leave the hospital rather than voicing their concerns to the healthcare team.

Interdisciplinary teamwork is one of the defining features of palliative care, and effective teamwork may reduce the risk of burnout among healthcare staff who provide palliative care [76]. The new MSF palliative care guidelines recommend that palliative and end-of-life care related decisions be made by a multidisciplinary team in discussion with the patient and family [34]. This approach to decision-making is important to ensure that all staff feel their concerns have been considered and no one bears the weight of ethically complex decisions alone. Yet unequal power between health professionals, rigid hierarchies, and lack of attention to the role of nurses are known barriers to effective team work in palliative care [77]. MSF struggles to disentangle itself from the colonial legacies that structure power differentials between international “expatriate” staff, locally hired “national staff”, and communities [44]. While there were examples in this study of national staff who appreciated palliative care related guidance offered by international staff, there were also times when they felt unable to challenge the decisions that international staff made. In contrast with some other MSF projects where national staff may have years or even decades of experience, at Goyalmara Hospital very high staff turnover meant that many doctors and nurses had limited pediatric or neonatal clinical experience and only a minority had been employed with MSF since the beginning of the 2017 Rohingya Refugee Crisis. For this reason, international staff pediatricians and some nurse managers were accurately perceived as having greater pediatric expertise. It is important to note; however, that in North America and Europe, pediatric and neonatal mortality rates do not approach the rates seen by staff at Goyalmara Hospital, and both international pediatricians acknowledged having limited palliative care related experience prior to their arrival in the project. Additionally, while international humanitarian staff may have content expertise, they do not have context specific knowledge [78] so critical to palliative care.

The task of dismantling unjust power differentials and creating an environment conducive to open discussions is complex; however, based on our findings and experience acting on these findings in practice, we would make several suggestions. Firstly, the interviews and FGDs that were conducted as part of this study provided valuable opportunities for staff to express their concerns in ways that did not always happen in routine clinical conversations. The act of conducting interviews and FGDs made it clear to staff that their views were valued, and offered dedicated time, space, and privacy for staff to express themselves. In order to center the role of non-medical staff with contextual knowledge and improve communication with families, we intentionally selected a health promotion team focal point to take leadership in coordinating care for children in palliative care. Based on this study, we would recommend clarifying the important roles that non-physician staff play in care planning discussions including their role in elucidating family values and preferences and communicating these with the rest of the healthcare team. Role clarification and training are needed to build staff confidence to authentically engage in care planning discussions, particularly among non-physician staff [79]. On an institutional level, during the spring of 2023, MSF UK began the recruitment process for a palliative care transformation project aimed at developing a user-friendly toolkit to support teams to integrate palliative care [80]. The project will be led by a nurse and public health professional and prioritizes an interdisciplinary approach to palliative care.

### Guidelines and palliative care decision-making

An important finding of this study was the way in which the authority of international staff intersected with the staff’s use and interpretation of palliative guidelines. For a number of reasons, including the urgency of many humanitarian interventions, MSF medical programs tend to be highly protocolized [81, 82]. Vandenberghe and Véran explain that within MSF, protocols can at times “become ends in themselves” and “are followed and implemented ‘ritualistically’” [82], descriptions that are reminiscent of our study findings. A qualitative study conducted in South Sudan and Bangladesh involving MSF and other humanitarian field workers found that inflexibility of policies and protocols was one source of ethical tension that staff experienced [81]. As we found in this study, even when clinical guidelines are designed to support rather than control practice, they may be operationalized and understood by field staff to be less flexible than intended. The 2021 MSF OCBA palliative care guidelines are clear in articulating the importance of local adaptation and individualized care planning [34], yet these and earlier palliative care-related guidelines were often understood by staff to be fairly rigid. Our findings suggest that the frequent rotation of international staff in positions of authority and inconsistent mentoring may have contributed to this rigid interpretation of palliative care guidelines. Both national and international staff felt it was important to consistently follow guidelines as a way of coping with international staff transitions. A major factor contributing to this inconsistent mentoring was likely a lack of palliative care specific training among international staff, as they reported in interviews, and the fact that they received their health professional training in regions of the world with disparate levels of palliative care awareness and integration [35]. MSF affiliated co-authors have explored the possibility of pre-departure palliative care training for pediatricians and nurse managers intending to work in projects with a palliative care focus.

Within MSF, there is an ongoing tension between the context-specificity and individuality of palliative care, and institutional efforts to create a replicable package of care to achieve our aim of expanding access to palliative care across MSF projects. While guidelines and protocols have clear value, they cannot eliminate uncertainty in palliative care related decision-making, particularly in the ethically complex situations that characterize humanitarian palliative care. Palliative care plans must be developed based on patient and family preferences and priorities so there is no single correct course of action that can be defined in a guideline. As a result, some degree of moral stress, a normal response to moral challenges [8], is likely unavoidable and an overreliance on protocols may even exacerbate staff moral distress. Given the complexity of palliative care related decision-making it is critical that moral distress is not left unaddressed as it may hamper staff’s ethical judgment and ability to act ethically in uncertain circumstances [8].

### Strengths and limitations

Several strengths of this study increase the trustworthiness of the findings. The ethnographic approach and prolonged period of fieldwork enabled a rich exploration of the moral experiences of staff in a particular local context. Given the single research site, we are not able to make definitive recommendations for program design in other contexts; however, the findings raise important questions and suggest areas of reflection for those involved in integrating palliative care in humanitarian contexts. An important strength and limitation of this study was the highly embedded role of the primary researcher (RY). Her identity as an international staff nurse undoubtedly influenced what staff shared during interviews and focus groups, but this was mitigated by her prolonged presence in the field and positive relationships with staff, as evidenced by their willingness to candidly share critique in their interactions with her. It is unlikely that her presence impacted staff behavior during participant observation beyond the usual impact of international staff presence given the prolonged period of fieldwork and since field notes were not prepared in the clinical departments. Given RY’s multiple roles, she had limited time to spend preparing field notes which led to a greater reliance on focus group and interview data in this study. While engaging multiple researchers in participant observation and data coding may have reduced the impact of a single researcher’s perspective, this limitation was mitigated through reflexive analytic discussions with the research team at each stage of the study. Since the study was focused on staff moral experiences, the findings do not reflect the experiences of patients or families.

### Recommendations for policy, practice, and research

Based on our findings, we have the following recommendations for practitioners and policymakers involved in integrating palliative care in humanitarian contexts: 1) take seriously the role of religion and spirituality for both patients, families, and staff and explore underlying values that may be consistent across the team, 2) focus on framing palliative as active care to reduce suffering and ensure that staff have the skills and tools necessary to intervene on behalf of their patients, 3) create space for open discussion and expression of concerns, paying particular attention to challenging questions and voices that are silent, and 4) ensure that palliative care guidelines emphasize individualized care planning which is informed by patient and family values and priorities, and that guidelines are used to support rather than discourage discussion. As evidence concerning the importance of palliative care in humanitarian contexts grows, there is a need for research on effective models for integrating palliative care, particularly in acute humanitarian emergencies where the challenges faced by staff at Goyalmara may be amplified. There is also a need for research exploring patient, family, and community experiences of palliative care interventions in humanitarian settings.

## Supporting information

S1 Appendix Goyalmara hospital palliative care training curriculum. (DOCX)

S2 Appendix Individual interview guide. (DOCX)

S3 Appendix Focus group discussion guide. (DOCX)

S1 Checklist Inclusivity in global research. (DOCX)
