# Correction: Doing our best and doing no harm: A focused ethnography of staff moral experiences of providing palliative care at a Médecins Sans Frontières pediatric hospital in Cox’s Bazar, Bangladesh

**DOI:** 10.1371/journal.pone.0298091

**Published:** 2024-01-25

**Authors:** Rachel Yantzi, Md Hadiuzzaman, Pradip Kumar Sen Gupta, Amin Lamrous, Kathryn Richardson, John Pringle, Lisa Schwartz, Puspita Hossain, David Kizito, Sakib Burza

There are errors in the Funding and Competing Interests statements. The correct statements are:

**Funding:** This study was funded by MSF-Spain using internal budgets dedicated to routine operational implementation. MSF provided support in the form of salaries for the following authors: RY, MH, AL, KR, JP, DK, and SB. The specific roles of these authors are articulated in the ‘author contributions’ section. MSF fulfilled the role of sponsor-investigator for this study, and as such was involved with the study design, data collection and analysis, decision to publish, and preparation of the manuscript.

**Competing Interests:** The authors have read the journal’s policy and have the following competing interests to declare: LS is a member of the MSF Ethics Review Board; however, she was not involved with the review of this protocol. This does not alter our adherence to *PLOS ONE* policies on sharing data and materials. There are no patents, products in development or marketed products associated with this research to declare.
