# Supplementary material for: Doing our best and doing no harm: A focused ethnography of staff moral experiences of providing palliative care at a Médecins Sans Frontières pediatric hospital in Cox’s Bazar, Bangladesh
Source: PLoS One. 2023 Jul 20;18(7):e0288938. doi: 10.1371/journal.pone.0288938 (PMC10358957; doi:10.1371/journal.pone.0288938)
Supplement: S1 Appendix — (DOCX) [file pone.0288938.s001.docx]

**Goyalmara Hospital Palliative Care Training Curriculum: July-August 2021**

|  | Professional Group | | | | | |
| --- | --- | --- | --- | --- | --- | --- |
| Topic | Palliative care focal points from each team including doctors, nurses, health assistants, mental health, health promotion and translators | Doctors | Nurses | Health Assistants | Mental Health Team | Health Promotion Team |
| Introduction to palliative care: Scope, patient populations, active care to relieve suffering |  |  |  |  |  |  |
| Basic symptom assessment and management (pain, dyspnea, nausea) |  |  |  |  |  |  |
| Opioid administration |  |  |  |  |  |  |
| Basic psycho-social and spiritual support |  |  |  |  |  |  |
| Communication and breaking bad news |  |  |  |  |  |  |
| Palliative care planning documentation |  |  |  |  |  |  |
| Community-based follow-up |  |  |  |  |  |  |
| End of life care, grief, and bereavement |  |  |  |  |  |  |
| Do-not-resuscitate order decision-making and documentation |  |  |  |  |  |  |

** In August 2021, we were anticipating the results of a large Ministry of Health nursing recruitment in the following 1-2 months which meant of a loss of more than 50% of our nursing staff. Therefore, we decided to focus training efforts on a small number of nurse focal points who planned to remain at Goyalmara. Short trainings were offered at the bedside to all nursing staff about opioid administration and DNR decision-making and documentation.
