# Supplementary material for: Doing our best and doing no harm: A focused ethnography of staff moral experiences of providing palliative care at a Médecins Sans Frontières pediatric hospital in Cox’s Bazar, Bangladesh
Source: PLoS One. 2023 Jul 20;18(7):e0288938. doi: 10.1371/journal.pone.0288938 (PMC10358957; doi:10.1371/journal.pone.0288938)
Supplement: S2 Appendix — (DOCX) [file pone.0288938.s002.docx]

Individual Interview Guide

| Main Questions | Follow-up Questions and Probes |
| --- | --- |
| - What does palliative care mean to you? - How is palliative care different or similar to end of life care?   After person describes and explores their definition:  “When we talk about end of life care, we are talking about care for patients who have only hours or days to live. When we talk about palliative care we mean care for children with life-threatening or life-limiting illness. The care is focused on reducing physical, emotional, social and spiritual suffering.” | - Examples of patients that would benefit from palliative or EOL care - What are the strengths or benefits of palliative care? - What are your fears or concerns about palliative care? - If you think of what you have been taught in palliative care trainings, are there differences in palliative care as you see it in practice? |
| - What does ‘good’ end of life care look like? - How do you think we could improve end of life care at Goyalmara Hospital? | - If you had a family member who was dying, what kind of care would you like them to receive? - What kind of care would you want to receive as a family member? - What helps you to be able to provide good end of life care? - What gets in the way of you providing good end of life care? - [If refers to psychosocial or mental support]: What does that look like? What do you mean by psycho-social support? How do you offer psycho-social support? |
| - How has COVID-19 affected how you care for patients and their families at end of life? |  |
| We have noticed that many families want to go home soon after learning that their child is likely to die (palliative discharges).   - How do you feel about these palliative discharges? - Can you help us understand the motivations behind this? - How can we better support these families, either to go home or stay with better support? | - Thoughts on palliative care discharges |
| - There are certain patients we care for at end of life that are hard to forget. Can you tell me the story of a patient or a situation that you found especially rewarding or meaningful? - Can you tell me about a situation or interaction here at the hospital that you found particularly difficult or troubling? | - How did you feel in that moment? - What did that mean to you? - What was most important to you in that moment? - How did the patient/family react? - If you were to do it all again, would you do anything differently? |
| - End of life care can involve a lot of decisions, sometimes very difficult decisions. Can you tell me about a recent end of life decision you witnessed or were involved in? | - - How were you involved in that decision?   - Who else was involved? How were they involved?   - How did you feel about the process?   - How was the decision made?   - What would have been helpful to you? |
| - How has your work providing end of life care impacted you personally? | - - What kinds of support do you think would be helpful?   - How do you cope with strong emotions like grief, sadness? |
