# Supplementary material for: Doing our best and doing no harm: A focused ethnography of staff moral experiences of providing palliative care at a Médecins Sans Frontières pediatric hospital in Cox’s Bazar, Bangladesh
Source: PLoS One. 2023 Jul 20;18(7):e0288938. doi: 10.1371/journal.pone.0288938 (PMC10358957; doi:10.1371/journal.pone.0288938)
Supplement: S3 Appendix — (DOCX) [file pone.0288938.s003.docx]

Focus Group Discussion Guide

| Main Questions | Follow-up Questions and Probes |
| --- | --- |
| - To begin, what comes to mind when we say, ‘end of life care’? Or palliative care? - After group shares and explores their definition:   “When we talk about end of life care, we are talking about care for patients who have only hours or days to live. When we talk about palliative care, we mean care for children with life-threatening or life-limiting illness. The care is focused on reducing physical, emotional, social and spiritual suffering.” |  |
| - What is most important at the end of life? - How would you describe a ‘good death’? | [If refers to psychosocial or mental support]: What does that look like? What do you mean by psycho-social support? How do you offer psycho-social support? |
| What is the role of [nurses, psycho-social staff, physicians etc.] in caring for patients who are dying? What about for their family members? |  |
| During your [medical/nursing/other] training, what were you taught about death and dying? |  |
| Can you tell me about any training or support have you received here at [HOSPITAL] or from MSF related to end-of-life care? |  |
| I want to talk a little bit about your experiences as a team of resuscitating patients using CPR. By CPR I mean rescue breathing and chest compressions. Can you tell me about a time when you as a team performed CPR and afterwards you felt good about what happened? | - Can you tell me about a time when you as a team performed CPR and you found the experience troubling or difficult? - What was different about that experience? - In what kinds of situations do you think CPR should not be provided? - How do you as a team decide that CPR should not be provided to a patient? - How does everyone feel about making those kinds of decisions? What are some of the benefits or challenges? |
| - We have noticed that many families want to go home soon after learning that their child is likely to die (palliative discharges). How do you feel about these palliative discharges? | - Can you help us understand why these families choose to leave? - How can we better support these families, either to go home or stay? |
| - How is the experience of providing end of life care different when the patient is Rohingya or from the host community? | - How do you feel about that? - Why do you think these differences exist? |
| - What do you think is needed improve end of life care at Goyalmara Hospital? |  |
